# Characterization and antitumor efficacy of poly(L-lactid acid)-based etoposide-loaded implants

**DOI:** 10.1080/10717544.2017.1321063

**Published:** 2017-05-05

**Authors:** Li Gao, Chuanqi Xie, Yuzhi Du, Xiaodong Wang, Erkang Xuan, Xiuxiu Liu, Yang Zhao, Jianjian Xu, Lan Luo

**Affiliations:** 1 State Key Laboratory of Pharmaceutical Biotechnology, School of Life Sciences, Nanjing University, Nanjing, People’s Republic of China,; 2 School of Biological and Medical Engineering, Hefei University of Technology, Hefei, People’s Republic of China,; 3 Laboratory of pharmaceutical research, Anhui Zhongren Science and Technology Co., Ltd, Hefei, People’s Republic of China, and; 4 Department of Pathology, The Second People’s Hospital of Hefei, Hefei, People’s Republic of China

**Keywords:** Etoposide, implant, poly(L-lactid acid), sustained release, intratumoral chemotherapy

## Abstract

Etoposide is widely used in the chemotherapy of a variety of malignancies. But the strong lipophilicity, poor bioavailability, and severe side effects of etoposide limit its clinical application. The aim of this study was to develop sustained-release etoposide-loaded implants and evaluate antitumor activity of the implants after intratumoral implantation. We prepared the implants containing etoposide, poly(L-lactid acid) and polyethylene glycol 4000 by the direct compression method. The implants were characterized regarding drug-excipient compatibility, content uniformity, morphology, sterility, *in vitro*, and *in vivo* release profiles. Then the antitumor activity of the implants was tested in xenograft model of A549 human non-small cell lung cancer. SEM images displayed smooth surface of the implant and indicated that etoposide was homogeneously dispersed in the polymeric matrix. The results of content uniformity met the requirements of the Chinese Pharmacopoeia. Both *in vitro* and *in vivo* release profiles of the implants were characterized by high burst release followed by sustained release of etoposide. Intratumoral implantation of etoposide-loaded implants could efficiently delay the tumor growth. Furthermore, increasing the dose of implants led to higher tumor suppression rate without adding systemic toxicity. These results indicated that etoposide-loaded implants have significant antitumor efficacy in xenograft model without dose-limiting side effects and they possess a strong potential to be used as an intratumoral chemotherapy option for lung cancer treatment.

## Introduction

Cancer is an evident public health problem worldwide and it is the second leading cause of death in the United States (Siegel et al., 2016). Cancer has also been the leading cause of death since 2010 in China (Chen et al., 2016). The WHO has estimated that 27 million cancer incidences and 17 million cancer deaths will occur by the year 2030 (Solano et al., 2013). Systemic chemotherapy is the most commonly used methods of cancer treatment. However, intravenously administered anticancer drugs must overcome transport barriers created by the high tumor interstitial fluid pressure before reaching the lesion side (Heldin et al., 2004). As a result, only a small fraction of the administered drugs could access the tumor site. On the other hand, higher systemic doses can result in undesirable side effects to normal tissues (Saltzman & Fung, 1997).

Local chemotherapy is considered as an alternative of conventional anticancer treatment where anticancer drugs are released directly at the tumor site. A wide range of materials can be used as reservoirs of anticancer drugs for local chemotherapy. These materials include poly(ethylene glycol) and its copolymers, polyure-thanes, poly(lactic acid) and its copolymers, poly(ɛ-caprolactone), polyanhydrides, chitosan, cellulose, cyclodextrins, silk, conducting polymers, modified titanium surfaces, calcium phosphate-based biomaterials, silicone, and silica implants, as well as carbon nanotubes and graphene (Saltzman & Fung, 1997; Wolinsky et al., 2012; Krukiewicz & Zak, 2016).

The development of polymer-based drug delivery systems that target therapy specifically at the tumor site can greatly improve antitumor efficacy and minimize systemic side effects (Weinberg et al., 2008). The local drug delivery strategies involving microspheres, nanoparticles, gels, polymeric rods, films, and wafers can be classified into intravenous administration system and local delivery system. The intravenous administration system consists of nano-materials which target the tumor tissues by passive diffusion or active targeting. The local delivery systems are administered intratumorally or coincide with tumor excision surgery with the ability to release the loaded drug for a prolonged period of time (Wolinsky et al., 2012).

Local drug delivery system provides a continuous sustained release of anticancer drugs and enables high drug concentrations at the target site, while reducing systemic toxicity. Several implantable sustained-release anticancer drugs have been commercially available and had a great success in clinic. Gliadel® (MGI pharma/Easai pharmaceuticals) is the first locally delivered antitumor implant containing carmustine approved by the FDA to treat recurrent malignant glioma in the USA. Other commercially available devices include Decapeptyl®, Lupron Depot®, Zoladex®, Eligard®, Viadur®, OncoGel®, and InGell® Delta (Saltzman & Fung, 1997; Krukiewicz & Zak, 2016). Furthermore, Sinofuan® (fluorouracial implants) have been widely used in peritoneal interstitial chemotherapy to treat alimentary system cancers in China (Shen et al., 2016).

Etoposide (VP16) is the first semi-synthetic topoisomerase II inhibiting anticancer agent derived from podophyllotoxin and approved for use by the FDA in the USA in 1983. Clinical trials demonstrated antitumor activity of etoposide in acute myeloid leukemia, Hodgkin’s disease, non-Hodgkin’s lymphoma, small cell lung cancer, non-small cell lung cancer, gastric cancer, breast cancer, and ovarian cancer (Hande, 1998). Now there are two commercial products of etoposide on the market: etoposide injections and oral soft capsules (Dong et al., 2013). However, both of these formulations have disadvantages. The low aqueous solubility of etoposide prevents its intravenous administration. Excipients used for etoposide injections such as ethanol, benzyl alcolhol, polysorbate 80, and polyethylene glycol are related to hypersensitivity reactions. Moreover, the oral administration of etoposide capsules exhibits a low bioavailability along with high inter- and intra- patient variability (Strickley, 2004; Solano et al., 2013). In addition, etoposide-related secondary leukemia has also been reported when it was used to treat lung cancer, non-Hodgkin lymphoma, neuroblastoma, acute lymphoid leukemia, Wilms tumor, and rhabdomyosarcoma (Ezoe, 2012). The strong lipophilicity and chemical instability limit its clinical application. Additionally, the conventional continuous intravenous infusion over 24–34 h is inconvenient to practice and causes pain to patients (Athawale et al., 2014) . Recently, many drug delivery systems have been developed with various pharmaceutical and pharmacological strategies to overcome the limitations of etoposide and have shown enhanced antitumor efficacy (Najar & Johri, 2014), including etoposide-loaded micoremulsions (Dong et al., 2013), polymer particles (Tang et al., 2010), micelles (Ukawala et al., 2012; Varshosaz et al., 2014; Chen et al., 2015), liposomes (Maswadeh et al., 2015; Skalickova et al., 2016), nanoparticles (Athawale et al., 2014; Wang et al., 2015b; Zhang et al., 2016) and etoposide-loaded poly (ɛ-caprolactone) implants (Solano et al., 2013).

In this study, we fabricated etoposide-loaded implants using poly (L-lactid acid) (PLLA) as main polymer matrix by the direct compression method. Furthermore, the etoposide-loaded implants were characterized in terms of content uniformity, morphology, sterility, *in vitro* and *in vivo* drug release from the implants. Further, we showed that intratumoral delivery of etoposide-loaded implants exhibited significant antitumor efficacy in A549 human non-small cell lung cancer xenograft model in nude mice.

## Materials and methods

### Chemicals and animals

Etoposide (purity ≥98%) was purchased from Sigma-Aldrich (St Louis, MO). Poly (L-lactid acid) (PLLA) (Molecular Weight, Mw =17 087) was generously provided by Anhui Zhongren Science and Technology Co., Ltd. (Anhui, China). Polyethylene glycol 4000 (PEG4000) was purchased from Beijing Huiyou Chemical Co., Ltd. Etoposide injection was purchased from Jiangsu Hengrui Medicine Co., Ltd. (Jiangsu, China). HPLC-grade acetic acid and acetonitrile were purchased from Tedia Company, Inc. (Fairfield, OH). Fetal calf serum was from Hyclone (Logan, UT). Ultra-pure water was obtained in a milli-Q system from Millipore (Bedford, MA). All other chemicals were of analytical grade.

Male BALB/c nude mice were purchased from Shanghai Lingchang Biological Technology Co., Ltd. (Shanghai, China) and kept in specific pathogen free conditions. Throughout the experiment, all mice had access to sterilized food and filtered water ad libitum. And all animal protocols were approval by Ethics Committee in Animal Experimentation at Nanjing University (Nanjing, China) and complied with the guideline for Care and Use of Laboratory Animals.

### Drug-excipient compatibility evaluation

Drug-excipient compatibility evaluation was carried out using stress testing method following CFDA Guidelines at the early stage of the preparation of etoposide-loaded implants (Committee, 2015). Two samples were prepared: (1) 10 mg of PLLA mixed with 2 mg of etoposide and (2) 20 mg of etoposide mixed with 1 mg of PEG4000. As per the CFDA Guidelines, the samples were stored at different stress conditions for duration of 10 days: 60 °C in hot air oven, 25 °C/90% ± 5% relative humidity (RH) and exposure to artificial daylight fluorescent lamp (4500Lx ± 500Lx). The drug content was determined by high-performance liquid chromatography (HPLC) after 10 days of the storage.

### Preparation of sustained-release etoposide-loaded implants

The sustained-release etoposide-loaded implants were prepared as solid rod by direct compression method under sterile conditions. Briefly, the dry powders containing 40% etoposide, 50% PLLA, and 10% PEG4000 (w/w) were sieved and fully mechanically blended for 25 s. The mixture were further molded into cylindrical implant. The blank implants were prepared similarly with the absence of etoposide.

### Characterization of etoposide-loaded implants

#### Scanning electron microscopy (SEM)

The implants were imaged using TM3000 tabletop scanning electron microscope (HITACHI, Tokyo Japan) to characterize the external and internal morphology. Prior to imaging, all samples were placed on metal sample holders and coated with gold. The surface and the cross-section morphologies of the implants were visualized at magnification of 600 × and 3000×. Images were obtained at 15 kV accelerating voltage.

#### Content uniformity of the etoposide-loaded implants

To determine the content uniformity of the implants, 10 implants were selected and weighed. Determination of content uniformity of the etoposide in the implants was performed according to the method stated in the Pharmacopoeia of the People’s Republic of China (Committee, 2015). Briefly, each implant sample was grounded with a pestle and mortar and dissolved in a mixture of acetic acid (pH 4.0) and acetonitrile (70:30, v/v). The residue was further dissolved in an ultrasonic water bath for 20 min. The resulting suspension was centrifuged at 12 000 rpm for 10 min. Subsequently, an aliquot of the supernatant (20 μl) was analyzed by HPLC. The actual drug content and the relative drug content of each implant were then calculated.

To evaluate the drug content uniformity, the acceptance value (AV) was calculated by the formula: AV= |100-X| + 2.2 S, where X is the mean of individual contents expressed as percentage of the label claim and S is the sample standard deviation. According to the standards of the Pharmacopoeia of the People’s Republic of China, the maximum allowed AV value is set to 15 (Committee, 2015).

#### *In vitro* release assay

The *in vitro* release assay was performed using the rotating basket method on a dissolution apparatus. Fifty milligram implants were placed in 300 ml release medium consisting of phosphate-buffered saline (PBS pH5.0) and isopropyl alcohol (93:7, v/v). The rotating speed of the basket was set at 130 rpm and the temperature of the release medium was maintained at 37 °C ± 0.5 °C. At predetermined time points, 3 ml of the sample was withdrawn, filtered and stored at 4 °C until HPLC assay. Then 3 ml of fresh release medium was added back to the dissolution flask to maintain a constant sink condition. The measurement was performed in triplicate for each batch.

#### Sterilization of sustained-release etoposide-loaded implants

The etoposide-loaded implants were sterilized using Co-60 gamma irradiation at a dose of 25 KGy. The membrane filtration method was used to test sterility and verification test was carried out using *staphylococcus aureus, Pseudomonas aeruginosa, Bacillus subtilis, Clostridium sporogenes, Candida albicans* and *Aspergillus niger* as positive control according to the guideline described in the Pharmacopoeia of the People’s Republic of China. The inoculated media were incubated for up to 14 days and the microbial growth was examined every day (Committee, 2015).

#### Cell culture and human non-small cell lung cancer xenograft model

The human non-small cell lung cancer A549 cells were grown in Dulbecco’s Modified Eagle’s Medium containing 10% (v/v) fetal calf serum, penicillin (100 U/ml) and streptomycin (100 μg/ml). Cells were cultured in a humidified atmosphere of 5% CO_2_ and 95% air at 37 °C. The cells suspension was adjusted to 1 × 10^7^ cells/ml. Then, 1 × 10^6^ cells in 100 μl cell culture medium were injected subcutaneously into the armpit of right anterior limb of each mouse. The tumor was allowed to grow for approximately 15 days when the tumor volume reached about 0.2 cm^3^ before initiation of the *in vivo* studies.

#### *In vivo* release assay

The *in vivo* release assay of the etoposide-loaded implants was conducted by intratumorally implanting the implants into A549 xenograft nude mice. Three implants were weighed and implanted into the center of the tumor. At predetermined time points (days 1, 5, 10, 15, 20, 25, and 30), the mice were sacrificed and the implants were retrieved from the tumor tissue. Three animals were used at each time point. After drying the implants, the amount of residual drug were determined by HPLC. The *in vivo* cumulative release percentage of etoposide was calculated as follows:

Etoposide release percentage(%)   =initial etoposide amount-residual etoposide amountinitial etoposide amount×100%

#### The HPLC method for determination of etoposide content in implants

The HPLC system (Shimadzu, Japan) was equipped with two LC-15 C pumps, a SPD-15 C essential UV detector and a CTO-15 C essential column oven. A Hypersil BDS C6H5 column (250 mm × 4.6 mm, 5 μm particle size) was used as an analytical column and maintained at 25 °C in the column oven. The mixture of acetic acid (0.2%, pH 4.0) and acetonitrile (70:30, v/v) was used as mobile phase and the flow rate was 1.5 ml/min. The injection volume was 20 μl and UV detection was performed at 254 nm. The external standard method was used for quantitative analysis.

### *In vivo* antitumor activity

Fifty male BALB/c-nu mice weighing approximately 18 g were used in the evaluation of antitumor activity. Subcutaneous tumors were inoculated in the nude mice as described above. In general, the mice bearing A549 tumor were randomly divided into five groups (*n* = 10 per group). Negative control group received single intratumoral implantation of blank implants (bland implant group). Positive control group received intraperitoneal injections of etoposide solution (VP16 solution group) at the dose of 25 mg/kg for three consecutive days (total drug content 1.5 mg). The doses were given according to the clinical usage of etoposide injection and exploration test on tolerance of etoposide solution in A549 tumor bearing mice. Low-dose etoposide-loaded implants treated group received single intratumoral implantation of the implants containing 0.75 mg etoposide (VP16 implant-L group). Medium-dose etoposide-loaded implants treated group received single intratumoral implantation of the implants containing 1.5 mg etoposide (VP16 implant-M group). High-dose etoposide-loaded implants treated group received single intratumoral implantation of the implants containing 3 mg etoposide (VP16 implant-H group). The implants were inserted into the center of the tumor using the modified 17 gauge trochar provided by Anhui Zhongren Science and Technology Co., Ltd. (Anhui, China). The tumor volume were measured every other day using digital caliper and calculated by the formula V(cm^3^) = length × (width^2^)/2 (Dong et al., 2009). At the end of the study, the mice were sacrificed and the tumors were collected and weighed. Moreover, the tumor suppression rate (TSR) was calculated using the formula TSR = (1 – Wt/Wc) × 100%, where Wt and Wc indicated the mean final tumor weight of treated group and negative control group, respectively (Dong et al., 2013). When tumor volume exceeded 2.5 cm^3^ or body weight decreased more than 20%, it was considered as the humane endpoint.

### Histological evaluation

At predetermined time point, one mouse in each group was sacrificed and the tumors were isolated. The tumor tissues were fixed in neutral 10% formalin solution and then dehydrated in a graded ethanol series. The tissues were embedded in paraffin and sectioned at 5 μm thickness. Tissue slides were stained with hematoxylin and eosin for histological evaluation. The histology images were taken using an Olympus BX51 microscope system (Tokyo, Japan).

### Statistical analysis

Statistical analyses were performed using a one-way analysis of variance where *P* value of <0.05 were considered significant. All the data were analyzed using GraphPad Prism version 5.0 software (San Diego, CA). One-way ANOVA and Tukey’s multiple comparison test was used to compare the means of all the experimental groups.

## Results

### Drug-excipient compatibility test

To investigate the compatibility of drug and excipients, dry powders of etoposide-PLLA and etoposide-PEG4000 were blended in a certain proportion. Stress testing method was used to assess the compatibility of the drug and excipients. As presented in Table 1, the samples did not show any significant visual changes throughout the storage period. Additionally, the drug content were not significantly changed on day 10 after being stored under 60 °C, 25 °C/90% ± 5% RH and strong artificial daylight.

**Table 1.** Drug content of etoposide in different stressed conditions.

			60 °C		25 °C/90% ± 5% RH		(4500 ± 500) LX	
Sample	Drug/excipient	Physical change	Day 0	Day 10	Day 0	Day 10	Day 0	Day 10
Etoposide + PLLA	1:5	NO	15.66%	16.03%	15.52%	16.02%	14.67%	15.47%
Etoposide + PEG4000	20:1	NO	92.10%	93.27%	91.69%	90.09%	92.68%	94.27%

### Preparation of etoposide-loaded implants

**Figure 1.**
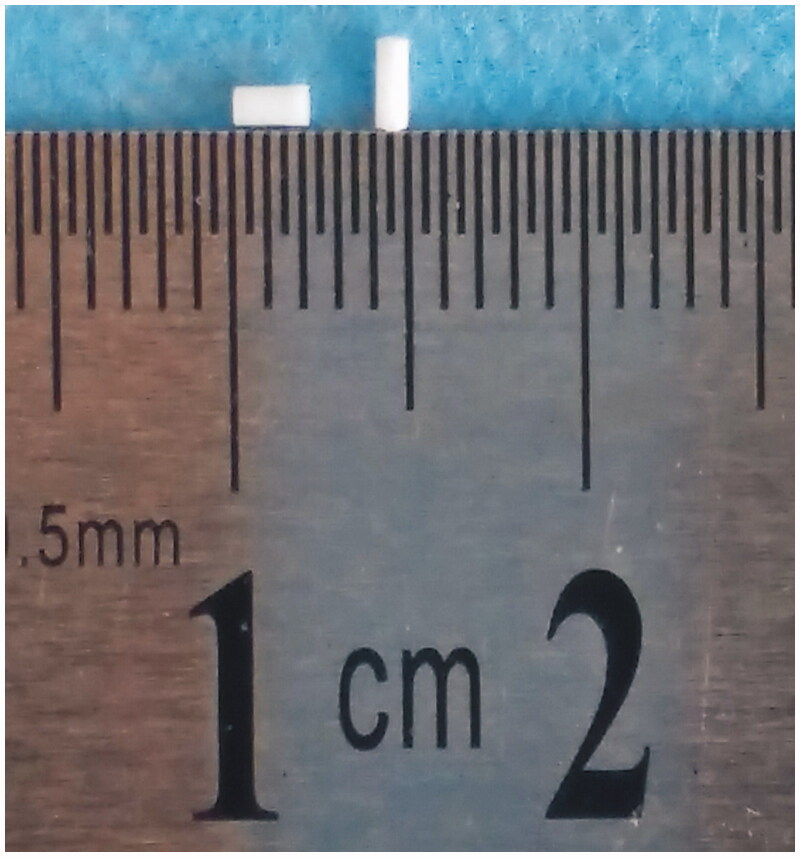
Macroscopic picture of the entire etoposide-loaded implants.

Dry powders of etoposide, PLLA and PEG4000 were mixed thoroughly and molded into cylindrical implants by direct compression method. The implants had an average weight of 1.33 ± 0.03 mg and an average length of 1.92 ± 0.14 mm (Figure 1). Moreover, the mean diameter of the implants was 0.9 mm (*n* = 6).

### Morphology of etoposide-loaded implant

**Figure 2.**
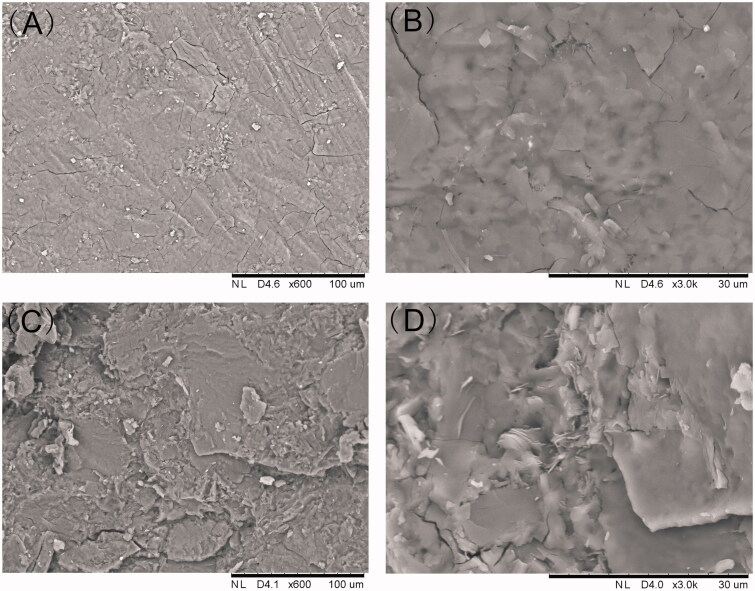
SEM picture of the etoposide-loaded implants. (A). External surface of the implant (magnification ×600). (B) External surface of the implant (magnification ×3000). (C). Cross-section of the implant (magnification ×600). (D). Cross-section of the implant (magnification ×3000).

SEM was used to evaluate the micromorphology of the implant which is an important characteristic for the drug release. As Figure 2 shown, the surface was found to be smooth and homogenous. Furthermore, the implant was cut with a scalpel to observe structure of the cross-section. The cross-section of the implant was a little rough in SEM but still homogenous without pores or channels.

### Content uniformity

To determine the content uniformity of the etoposide-loaded implants, ten implants were selected and tested the drug content by HPLC complied with the method described in the Pharmacopoeia of the People’s Republic of China. The mean value of actual drug content of the tested implants were assayed to be (37.84 ± 0.12)% and that was close to the label claim of the drug (40%, w/w). The mean value of relative drug content was (94.61 ± 0.29) %. According to the formula AV= |100-| + 2.2 S, the acceptance value (AV) of content uniformity was calculated to be 6.03 which was significantly lower than the maximum allowed acceptance value (15).

### *In vitro* and *in vivo* drug release from the implants

**Figure 3.**
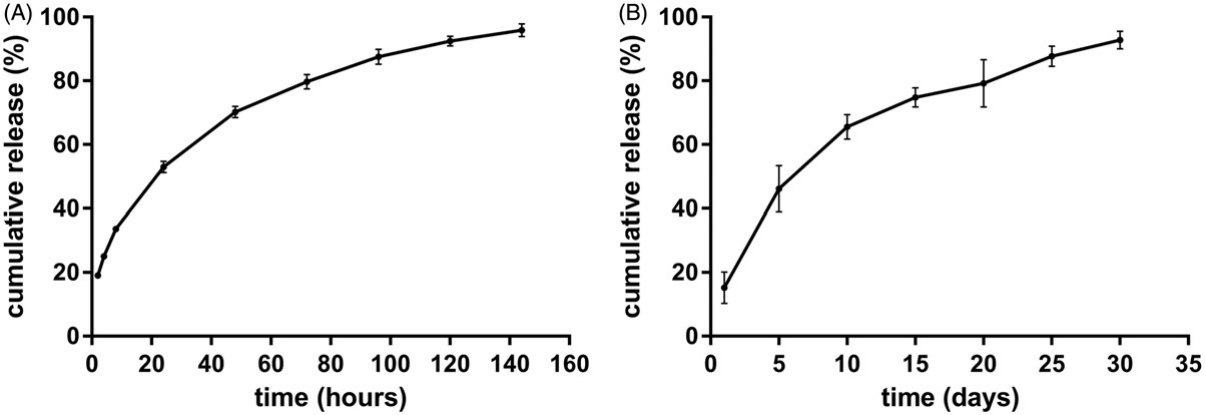
The release profiles of etoposide-loaded implants. (A) The *in vitro* cumulative release profiles of etoposide from the implants. Data are shown as mean ± standard deviation (*n* = 6 for each time). (B) The *in vivo* cumulative release profiles of etoposide from the implants. Data are shown as mean ± standard deviation (*n* = 6 for each time).

The *in vitro* cumulative release test was carried out in the release medium under suitable sink condition. The *in vitro* release profile was depicted in Figure 3(A). Approximately 20% of drug was released in the first 2 h. The mean cumulative release percentage was 52.9% in the first day. On day 2, 17.3% of etoposide was released from the implant. From day 3, the drug release rate gradually slowed down. As a whole, the cumulative release reached an average of 95.9% on day 6. To gain the information of the *in vivo* release profile, the etoposide-loaded implants were implanted intratumorally into A549 tumor bearing mice and then the implants were collected on day 5, 10, 15, 20, 25, and 30 post-implantation. The result was shown in Figure 3(B). The implants released 15.2% of drug on the first day and 46% of drug within 5 days. The mean cumulative release percentage reached 65.6% on day 10. Subsequently, the drug release slowed down and the implants released the drug almost at a constant rate. The *in vivo* release duration was as long as 30 days.

### Sterility test

Gamma radiation from ^60^CO was used to sterilize the etoposide-loaded implants. Exposure to gamma radiation at dose of 25 KGy caused no changes in the drug content of etoposide. Membrane filtration method was used for detection of microbial contaminants of the radiated implants. During a 14-day incubation period, microbial growth was not observed in any tube containing etoposide-loaded implants.

### *In vivo* antitumor efficacy

**Figure 4.**
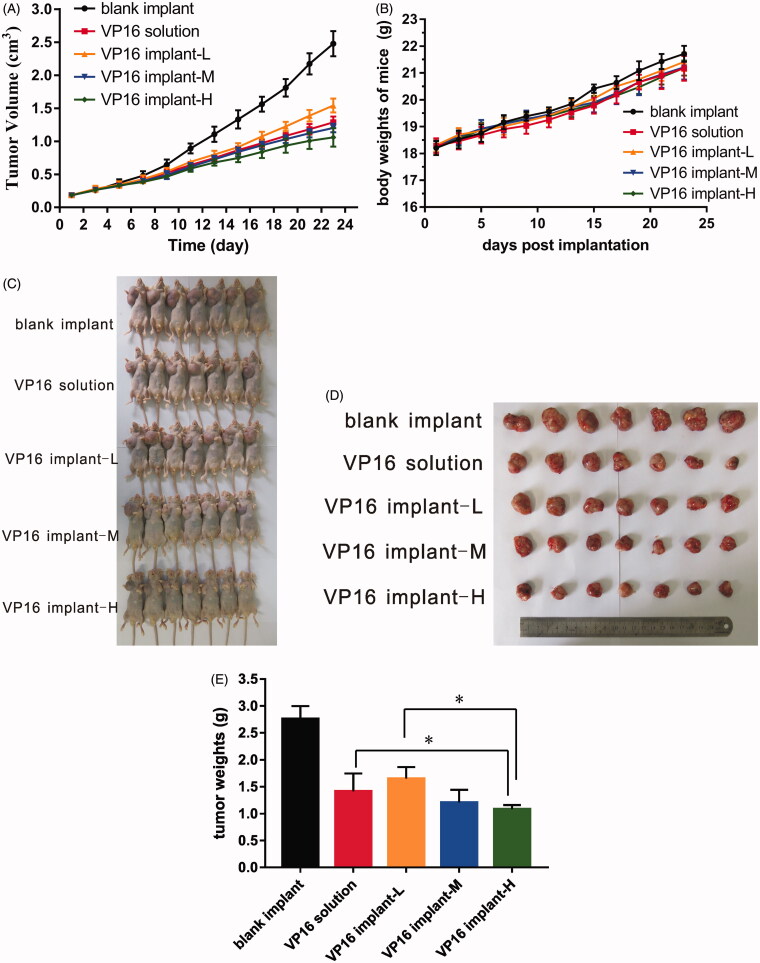
Antitumor efficacy of etoposide-loaded implants on A549 xenograft mouse model. (A) Tumor growth curve of the tumor-bearing mice after intraperitoneal administration of etoposide solution or implantation of different doses of etoposide-loaded implants. (B) The average body weight of mice during the treatment period. (C) Picture of the mice bearing A549 tumor on day 23 post implantation. (D) Picture of tumors dissected from mice on day 23 post-implantation. (E). The average tumor weight of each group (*P* value less than 0.05 was marked as *).

The evaluation of antitumor activity was conducted in BALB/c nude mice inoculated with A549 human non-small cell lung cancer cell lines. The tumor growth curve was presented in Figure 4(B). The tumor had grown rapidly in blank implant group and tumor size exceeded 2.5 cm^3^ on day 23 post implantation. Both etoposide solution and etoposide-loaded implants delayed tumor growth effectively. When the high-dose implants containing 3 mg etoposide were given, which is twice the therapeutic dose for human, we observed more significant tumor growth inhibition compared with other groups.

At the end of the experiment (day 23 post implantation), mice were sacrificed and tumors dissected from the mice were weighed. The mean final tumor weight of blank implant group was 2.76 ± 0.24 g and 1.41 ± 0.33 g in VP16 solution group. The mean final tumor weights were 1.65 ± 0.21 g and 1.21 ± 0.24 g for VP16 implant-L and VP16 implant-M groups, respectively. Furthermore, the mice in VP16 implant-H group had an average tumor weight of 1.08 ± 0.08 g (Figure 4E). As shown in Figure 4(E), the tumor weight of VP16 implant-H group was significant lower than VP16 solution and VP16 implant-L group. The value of TSR in all treated groups exceeded 40%. The value of TSR of VP16 implant-M group (56.3%) was greater than that in VP16 solution group (48.7%). The TSR value of VP16 implant-H group increased to 60.8% which was significantly greater than other groups. During the experiment period, the body weights of all mice increased slowly but there was no significant difference among the groups (Figure 4B). Furthermore, all the mice survived till the end of the experiment.

**Figure 5.**
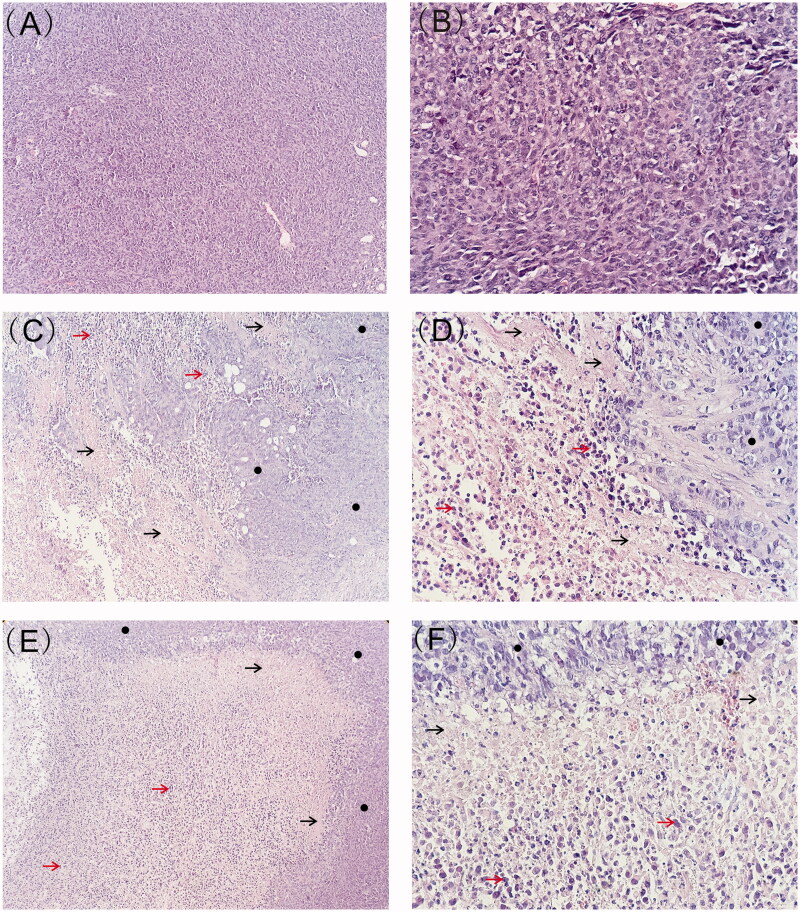
Typical histology images of tumors retrieved on day 7 and day 23 post-implantation (black arrow represents necrotic area, red arrow represents nuclear debris of tumor cells and black circle represents viable tumor cells). (A) Histology image of tumor treated with blank implants (magnification ×100). (B) Histology image of tumor treated with blank implants (magnification ×400). (C) Histology image of tumor treated with high-dose etoposide-loaded implants (drug content 3 mg) on day 7 post implantation (magnification ×100). (D) Histology image of tumor treated with high-dose etoposide-loaded implants (drug content 3 mg) on day 7 post implantation (magnification ×400). (E) Histology image of tumor treated with high-dose etoposide-loaded implants (drug content 3 mg) on day 23 post-implantation (magnification ×100). (F) Histology image of tumor treated with high-dose etoposide-loaded implants (drug content 3 mg) on day 23 post-implantation (magnification ×400).

Representative histological photographs of tumor tissue sections were presented in Figure 5. The tumor from blank implant group was filled with viable tumor cells while those from etoposide-loaded implants treated groups exhibited large necrotic areas mixed with cellular debris. After 7 days of implantation, the tumor tissue exhibited evident necrotic areas containing regions with nuclear debris of tumor cells. Larger areas of necrosis were observed 23 days after implantation. Moreover, the high-dose etoposide-loaded implants resulted in more extensive necrosis in tumor tissues.

## Discussion

Etoposide is a commonly used drug in the chemotherapy of a variety of malignancies. Moreover, the cisplatin-etoposide regimen has been one of the standard combination chemotherapy extensively used in the treatment of advanced non-small cell lung cancer (Ardizzoni et al., 1999; Arriagada et al., 2004). Systemic chemotherapy remains to be the primary treatment for lung cancer but the therapeutic effectiveness is often limited because of dose-limiting side effects (Tang et al., 2010). In addition, low aqueous solubility and poor/variable bioavailability of etoposide limit its clinical use. The activity of etoposide is highly schedule-dependent and prolonged exposure of etoposide to malignant cells can cause dose-dependent DNA breaks that would be expected to yield superior antitumor efficacy. Furthermore, removal of etoposide can leads to fast repair of DNA breakage. However, higher blood etoposide concentrations may result in side effects such as myelosuppression and treatment-related leukemia (Hande, 1996). A phase III randomized trial carried out by the North Central Cancer Treatment Group indicated that 72 h infusion of etoposide was associated with higher toxicity and did not show any superiority in response rate and survival as compared with bolus treatment (Ardizzoni et al., 1999). There is an urgent need to explore novel drug delivery system to overcome the limitations of existed formulations. In this study, we developed sustained-release etoposide-loaded implants directly targeting at tumor site and aimed to maximize the therapeutic index of etoposide while reducing the treatment-related side effects.

PLLA was the main excipient used in the fabrication of the etoposide-loaded implants. PLLA is a typical stereoisomer of PLA which is widely used in biomedical applications. In addition, PLLA is the most promising synthetic biodegradable polymers that has been approved by the FDA for implantable medical devices including bioresorbable scaffolds, sutures, dental devices, orthopedic plates and screws (Xu et al., 2011; Bergstrom & Hayman, 2016). Recently, PLLA has been used as a polymeric matrix in novel drug delivery systems (Loo et al., 2010; Liu et al., 2013; Wu et al., 2014; Gardella et al., 2016). PEG polymer was the other excipient of the implants which was characterized by low melting point, low toxicity, wide drug compatibility and hydrophobicity. PEG has been widely used as drug carrier and addition of PEG can facilitate the dissolution and release rate of the drug from implants by promoting the water diffusion into the implants (El-Badry et al., 2009; Cheng et al., 2010; Wang et al., 2015a).

The drug-excipient compatibility testing was conducted at an early stage of preparation of etoposide-loaded implants. HPLC is one of the non thermal methods used to detect compatibility (Liltorp et al., 2011). In this study, the samples were stored in different stress conditions for 10 days, and the drug content was determined using HPLC method. During the storage period, no morphological changes and significant changes in drug content were observed in drug-excipient combinations, indicating that the blend of etoposide, PLLA and PEG4000 met the requirements of the Pharmacopoeia of the People’s Republic of China.

Etoposide-loaded implants were prepared by direct compression of dry blends containing etoposide, PLLA and PEG4000. The direct compression method is most widely used in drug preparation without using of organic solvents which are often toxic to environment and patients (Kreye et al., 2011). Furthermore, it is considered as an efficient and economic method because it reduces processing time and manufacturing steps. Another benefit of the method is cost-savings because it requires less labor equipment (Upadhyay et al., 2014). The implants were molded into cylinder with the diameter of 0.9 mm because it is convenient to administer them by modified 17 gauge trochar.

The SEM images of the implants demonstrated the homogenous drug distribution in the formulation. Content uniformity testing is a pharmaceutical analysis parameter for the quality control of solid dosage. In this work, the acceptance value of content uniformity was 6.3 which met the requirements for content uniformity of the Pharmacopoeia of the People’s Republic of China (Committee, 2015). The result suggested that etoposide and the excipients were sufficiently mixed in the fabrication process and etoposide presented a uniform distribution in the polymer matrix.

The etoposide-loaded implants exhibited initial burst effect followed by sustained-release profiles both *in vitro* and *in vivo*. The burst release may be due to fast dissolution and diffusion of etoposide from the surface of the implants. The drug release rate gradually declined. This can be explained by the fact that both PLLA and etoposide are hydrophobic, so it is not easy for etoposide to be released from the PLLA and diffusion into the medium (Solano et al., 2013). After being implanted into the xenograft tumor, the duration of drug release became longer compared with *in vitro* settings. It is known that the optimal drug release profile is characterized by the ability to release large amount of drug early to reach the therapeutic concentration followed by sustained release to maintain the therapeutic concentration (Weinberg et al., 2008). The prolonged exposure of etoposide to cancerous cells will produce a longer period of enzyme inhibition and increase anticancer efficacy of etoposide that has been considered to be essential for the success of chemotherapy treatment (Solano et al., 2013).

In this study, we investigated the antitumor efficacy of etoposide-based implants using a human non-small cell lung cancer xenograft mouse model. It is observed that both intraperitoneal injection of etoposide solution and intratumoral implantation of etoposide-loaded implants delayed tumor growth efficiently. The total dose administered via the implants (drug content 1.5 mg) was equivalent to the total amount administered intraperitoneally. The measurement of tumor volume showed that animals received intratumoral implantation of implants containing 1.5 mg etoposide did not show much superior tumor growth inhibition compared to equivalent dose of free etoposide administration. The TSR value increased when higher doses of etoposide-loaded implants were given because a larger amount of etoposide released from the implants, accumulated in the tumor site and resulted in strong antitumor efficacy. It is interesting that escalating the drug content of etoposide-loaded implants to 3 mg elicited significant antitumor effect without additional toxicity. The tumor growth curve indicated that etoposide-loaded implants could inhibit tumor grow in a dose-dependent manner.

Histological evaluation of tumor tissues confirmed the antitumor activity of etoposide-loaded implants. Higher-dose implants resulted in more severe tumor cell destruction. It is worth noting that no infiltration of inflammatory cells was observed in the tumor tissue. Moreover, we did not find fibrous capsule formation around the implantation site. Inflammation reaction is the host response to the implanted biomaterial and the degree of the response depends on the properties of the implant, such as size, morphology, composition, stability, sterility, contact duration, and degradation (Hussein et al., 2016). An implant with good biocompatibility must not be recognized as foreign by immune system and induce inflammatory reactions (Bauquier et al., 2016). The histological studies revealed that PLLA-based etoposide-loaded implants have provided an acceptable histocompatibility after implantation into the tumor.

## Conclusion

In this study, we prepared PLLA based etoposide-loaded implants by direct compression method. The drug-excipient compatibility test showed that the blend of etoposide, PLLA and PEG4000 met the requirements of the Pharmacopoeia of the People’s Republic of China. The SEM results and content uniformity testing demonstrated that etoposide was homogeneously dispersed in the polymeric matrix. Both *in vitro* and *in vivo* release profiles of the implants were characterized by high burst release followed by sustained release of etoposide. The antitumor efficacy of etoposide-loaded implants conducted in nude mice bearing A549 cell line illustrated that etoposide-loaded implants had a significant antitumor activity on the A549 human non-small cell lung cancer xenograft model in nude mice. Furthermore, escalating the dose of implants resulted in higher antitumor effectiveness without additional systemic toxicity. We conclude that the PLLA based etoposide-loaded implants have the potential to be used as a new intratumoral chemotherapy method to treat lung cancer in humans.
